# Supplementary material for: OpenAnnotate: a web server to annotate the chromatin accessibility of genomic regions
Source: Nucleic Acids Res. 2021 May 17;49(W1):W483–90. doi: 10.1093/nar/gkab337 (PMC8262705; doi:10.1093/nar/gkab337)
Supplement: gkab337_Supplemental_File [file gkab337_supplemental_file.pdf]

OpenAnnotate: a web server to annotate the chromatin accessibility of genomic regions

Shengquan Chen[1], Qiao Liu[1], Xuejian Cui[1], Zhanying Feng[2,3], Chunquan Li[4], Xiaowo Wang[1], Xuegong Zhang[1], Yong Wang[2,3] and Rui Jiang[1,*]

[1] Ministry of Education Key Laboratory of Bioinformatics, Research Department of Bioinformatics at the Beijing National Research Center for Information Science and Technology, Center for Synthetic and Systems Biology, Department of Automation, Tsinghua University, Beijing 100084, China
[2] CEMS, NCMIS, MDIS, Academy of Mathematics and Systems Science, Chinese Academy of Sciences, Beijing 100190, China
[3] Center for Excellence in Animal Evolution and Genetics, Chinese Academy of Sciences, Kunming 650223, China
[4] School of Medical Informatics, Daqing Campus, Harbin Medical University, Daqing 163319, China

* To whom correspondence should be addressed. Tel: +86 10 6279 5578; Fax: +86 10 6278 6911; Email: ruijiang@tsinghua.edu.cn

Supplementary Notes

Supplementary Note S1. Parallel strategy and system design

The main challenge for annotating openness of massive genomic regions is querying reads and peaks at high frequencies in a vast amount of chromatin accessibility samples. To circumvent this issue, we designed a parallel strategy that endows OpenAnnotate with the ability to annotate openness efficiently. We used C++, a programming language with high efficiency, to develop a multithreaded program that consists of a reading module, a calculating module, and a writing module. As illustrated in Supplementary Figure S1, the reading module packages the genomic regions to be annotated into data blocks and pushes them into the input pipeline *I_pipe*. Each data block contains a subset of the genomic regions and the header information that includes the number and indexes of these regions in the entire set. When a thread in the calculating module is idle, the data block will be automatically extracted from the input pipeline for calculation. The results are pushed into the output pipeline *O_pipe* in a similar form of data blocks, which are then popped out and written into disks by the writing module. With the communication pipeline *C_pipe*, the reading module can respond to the working state of the writing module. When the writing speed is lower than the reading speed, the reading module will pause to push the data block into *I_pipe* to effectively save memory resources.

The current version of OpenAnnotate was deployed on a calculation node and a server node of a high-performance computing cluster. The calculation node has two Intel scalable processors (56 hyper-threads in total), 768 GB RAM, and 480 TB storage space. In addition, two 2TB Samsung 980 PRO SSDs, each of which can achieve sequential read/write speed up to 7000/5100 MB/s and random read/write speed of 1000K IOPS (queue depth 32 with 16 threads), were adopted to cache hard drives for supporting intensive reading and writing operations. The server node bridges web interfaces and the calculation node by receiving tasks from users and returning results or other information for visualization and downloading. Incron (http://inotify.aiken.cz/?section=incron) is used to monitor filesystem events and automatically execute predefined commands. Once the server node receives and saves a new task in a specific file path, the parallel program in the calculation node will be executed automatically, and the real-time results can be accessed from the web server. With the parallel strategy and system design, OpenAnnotate can provide real-time annotation results within about 10 seconds depending on the input file size and the internet speed, and annotate foreground read count, raw read openness, and ATACdb peak openness of 100 thousand genomic regions of size 150 base pairs in 1493 chromatin accessibility samples within 2 minutes and 32 minutes on the mode of region-based annotation and per-base pair annotation (15 million base pairs), respectively.

Supplementary Note S2. Cell type-specificity of human A549 enhancers

Following the analysis pipeline in SilencerDB, we introduce a basic application of OpenAnnotate to reveal the cell type-specificity of regulatory elements. Taking experimentally validated human A549 enhancers in

EnhancerAtlas 2.0 as an example, we aim to check whether the enhancers show higher degrees of chromatin accessibility in A549 than in other biosample types. We first used OpenAnnotate to annotate openness of the enhancers across 199 human DNase-seq biosample types, then performed one-sided Wilcoxon tests for the openness scores in A549 versus those in each of the remaining 198 biosample types, and finally obtained 198 FDR *p*-values (Benjamini and Hochberg correction). Results demonstrate that these A549 enhancers have higher openness scores in A549 than in all the other 198 biosample types (FDR=0.01), suggesting the cell line specificity of these enhancers.

Supplementary Note S3. Identification of regulatory elements and 3D chromatin contacts

DeepCAPE integrates DNA sequences and DNase-seq data to accurately predict enhancers. Specifically, a DNA sequence of length L is one-hot encoded as a $4 \times L$ matrix, and a $k \times L$ matrix containing raw read openness of each site in the sequence is obtained by OpenAnnotate using the *Per-base pair annotation* mode, where k denotes the number of replicates of a specific cell line and varies in different cell lines. These two matrices are fed into DeepCAPE to predict the probability that the DNA sequence is an enhancer. The adoption of auto-encoder provides an effective approach to reduce and unify the dimensionality of openness data in different cell lines, thus enabling the cross cell-line identification of enhancers. With the integration of openness data, DeepCAPE consistently outperformed existing methods, especially those only use DNA sequence data, for the identification of cell line-specific enhancers. Such machine learning frameworks can also be adapted for the prediction of other functional elements in the genome, including but not limited to promoters, silencers, insulators, and locus control regions.

Analogously, to predict 3D chromatin contacts, DeepTACT takes the one-hot encoded DNA sequences of two regulatory elements, and the corresponding per-base pair annotated raw read openness in a specific cell line as inputs. The output of DeepTACT is the probability that the two regulatory elements have 3D contact. DeepTACT outperformed existing methods on the task of inferring both promoter-enhancer and promoter-promoter interactions. Besides, the functional hub promoters and disease-related regulatory elements identified by DeepTACT indicate that the openness annotated by OpenAnnotate plays an important role in understanding regulatory mechanisms.

Supplementary Note S4. Single-cell chromatin accessibility sequencing data analyses

refProj contains the following major steps: (i) perform the term frequency-inverse document frequency (TF-IDF) transformation to normalize the scCAS data (a cell-by-peak matrix), (ii) annotate the bulk openness of peaks in the scCAS data by OpenAnnotate (referred as the reference data), and (iii) apply principal component analysis (PCA) on the reference data and use the learned first 30 projection vectors to project the TF-IDF transformed scCAS data matrix. The obtained low-dimensional representation of cells can then be applied to downstream analyses.

We annotated the openness of peaks of the Buenrostro2018 dataset using all 871 human DNase-seq samples, and then used raw read openness and narrow peak openness to implement refProj, respectively. We implemented UMAP visualization and performed cell clustering (hierarchical clustering, *k*-means

clustering and Louvain clustering) using the source code obtained from the benchmark study. We sorted the methods by maximum adjusted Rand index (ARI) score as the benchmark study. As shown in Figure 2B, refProj-peak (peak openness-based) and refProj-read (read openness-based) achieve the third-best and the sixth-best clustering performance, respectively. Among the top-six methods, SnapATAC, Cusanovich2018 and cisTopic were shown to be state-of-the-art methods in the benchmark study. Data visualization using the top-six methods further demonstrates the effectiveness of refProj to characterize cell heterogeneity (Figure 2C). We note that although Cusanovich2018 achieved the second-highest ARI using Louvain clustering, it hardly worked using hierarchical clustering and *k*-means clustering, indicating the insufficient robustness to clustering method. To assess the overall performance using different clustering methods, we sorted the scCAS analysis methods by average ARI score but not by maximum ARI score. The ranked top-six methods are cisTopic, SnapATAC, refProj-peak, refProj-read, chromVAR-kmers+PCA, and chromVAR-motifs, which again demonstrate that the openness annotated by OpenAnnotate can effectively facilitate the analysis of scCAS data.

Note that although refProj simply uses all biosample types as a reference without the selection of cell type-specific chromatin accessibility samples, it implicitly assumes that all the biological variation is shared in single-cell and the reference data, as it only uses the projection vectors learned from the reference bulk data and does not use single-cell data to learn the projection vectors. Therefore, statistical or machine learning methods can be developed to better characterize the scCAS data. For example, methods can be developed to simultaneously model (i) the shared biological variation among scCAS data and reference data, and (ii) the unique biological variation in scCAS data that identifies novel cell subpopulations.

Supplementary Figures

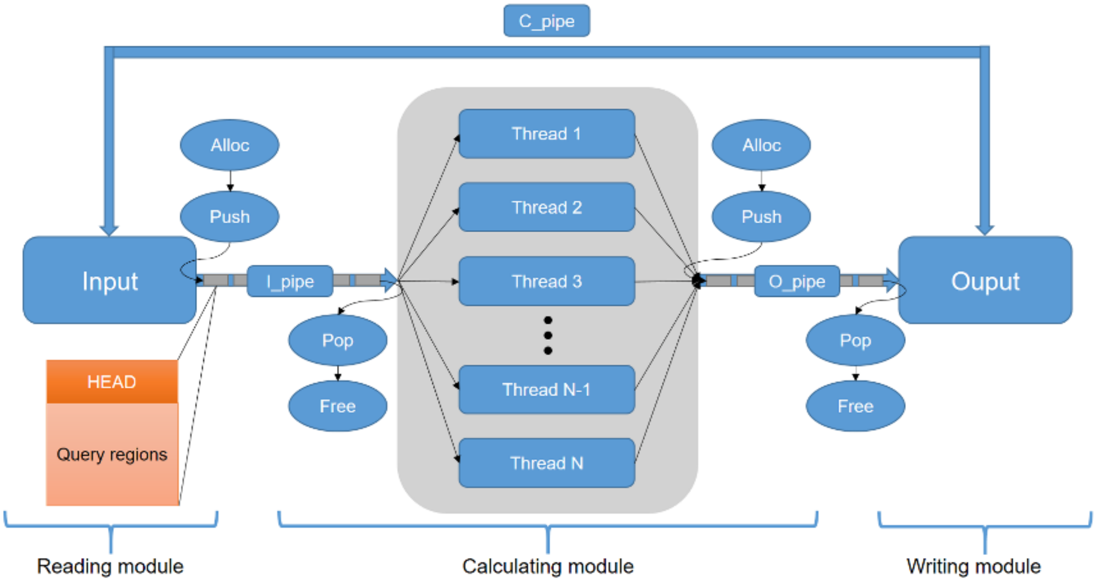

Supplementary Figure S1. The parallel strategy for efficiently annotating openness of massive genomic regions in a vast amount of chromatin accessibility samples.

Supplementary Tables

Supplementary Table S1. Comparison of data and functionalities in OpenAnnotate with other databases and toolkits.

Data and functionalities	OpenAnnotate	ATACdb	Cistrome DB/Toolkit	DeepBlue	ENCODE /SCREEN	Ensembl	GTRD	OCHROdb
Homo sapiens, DNase-seq	✓		✓	✓	✓	✓	✓	✓
Homo sapiens, ATAC-seq	✓	✓	✓	✓	✓		✓	
Mus musculus, DNase-seq	✓		✓	✓	✓	✓	✓	
Mus musculus, ATAC-seq	✓		✓		✓			
Hierarchical categorization	✓	✓		✓	✓			✓
Query a given genomic region	✓	✓	✓		✓	✓		✓
Comparison across biosample types	✓		✓		✓	✓		✓
Comparison across tissues	✓							
Comparison across biological systems	✓							
Annotation for batch genomic regions	✓							
Annotation in a specific biosample type	✓				✓	✓		
Per-base pair annotation	✓							
Ultra-efficient calculation	✓							
Standardized annotation results	✓							
Real-time browsing of massive results	✓							
Heatmap visualization of openness	✓							
Retrieve results by task ID	✓							
One-click archive to mailbox	✓							
MD5 to verify the download	✓							
Elaborate application notebooks	✓							
Applied to cell type-specific studies	✓							
Incorporated into computational models	✓							
Used as reference for single-cell analyses	✓							
